# Mucosal Immunity against Neuraminidase Prevents Influenza B Virus Transmission in Guinea Pigs

**DOI:** 10.1128/mBio.00560-19

**Published:** 2019-05-21

**Authors:** Meagan McMahon, Ericka Kirkpatrick, Daniel Stadlbauer, Shirin Strohmeier, Nicole M. Bouvier, Florian Krammer

**Affiliations:** a Department of Microbiology, Icahn School of Medicine at Mount Sinai, New York, New York, USA; b Graduate School of Biomedical Sciences, Icahn School of Medicine at Mount Sinai, New York, New York, USA; c Department of Biotechnology, University of Natural Resources and Life Sciences, Vienna, Austria; d Division of Infectious Diseases, Department of Medicine, Icahn School of Medicine at Mount Sinai, New York, New York, USA; St. Jude Children’s Research Hospital

**Keywords:** influenza, influenza B, neuraminidase, transmission, vaccine

## Abstract

Despite efforts to control influenza virus infection and transmission, influenza viruses still cause significant morbidity and mortality in the global human population each year. Most of the current vaccines target the immunodominant hemagglutinin surface glycoprotein of the virus. However, reduced severity of disease and viral shedding have also been linked to antibodies targeting the second viral surface glycoprotein, the neuraminidase. Importantly, antineuraminidase immunity was shown to be relatively broad, in contrast to vaccine-induced antibodies to the hemagglutinin head domain. In this study, we assessed recombinant neuraminidase protein vaccination for its ability to prevent or limit virus transmission. We vaccinated guinea pigs either intramuscularly or intranasally with a recombinant influenza B virus neuraminidase to assess whether neuraminidase vaccination via these routes could prevent transmission of the homologous virus to a naive recipient. Guinea pigs vaccinated with neuraminidase showed reduced virus titers; however, only vaccination via the intranasal route fully prevented virus transmission to naive animals. We found high levels of antineuraminidase antibodies capable of inhibiting neuraminidase enzymatic activity in the nasal washes of intranasally vaccinated animals, which may explain the observed differences in transmission. We also determined that mucosal immunity to neuraminidase impaired the transmission efficiency of a heterologous influenza B virus, although to a lesser extent. Finally, we found that neuraminidase-vaccinated animals were still susceptible to infection via the airborne and contact transmission routes. However, significantly lower virus titers were detected in these vaccinated recipients. In summary, our data suggest that supplementing vaccine formulations with neuraminidase and vaccinating via the intranasal route may broadly prevent transmission of influenza B viruses.

## Abstract (precis)

Recently, the protective effect of anti-neuraminidase immunity has been highlighted by several studies in humans and animal models. However, so far the role that anti-neuraminidase immunity plays in inhibition of virus transmission has not been explored. In addition, neuraminidase has been ignored as an antigen for influenza virus vaccines. We show here that neuraminidase-based vaccines can inhibit the transmission of influenza virus. Therefore, neuraminidase should be considered as an antigen for improved influenza virus vaccines that not only protect individuals from disease but also inhibit further spread of the virus in the population.

## INTRODUCTION

Current seasonal inactivated influenza virus vaccines focus on inducing antibody responses toward the major surface glycoprotein of the virus, the hemagglutinin (HA). These vaccines are only efficacious when well matched with circulating strains (1, 2). Unfortunately, over time, the HA accumulates amino acid changes at its antigenic sites, leading to antigenic drift that renders the previous year’s vaccines ineffective and forces the annual reformulation and readministration of influenza vaccines (2, 3). Recently, it has been shown that natural infection, but not vaccination, results in the generation of antibody responses that target the second surface glycoprotein, the immunosubdominant neuraminidase (NA) (4–6). The influenza virus NA is subject to reduced immune pressure which might contribute to slower amino acid changes in antigenic sites (7, 8). As such, anti-NA antibodies have been shown to broadly bind heterologous NAs and can protect mice from lethal influenza A virus challenge (4, 9). Hence, antibodies that target the NA have the potential to be broadly protective within influenza virus subtypes.

The viral NA is a tetrameric type II transmembrane protein that has receptor-destroying activity, which is integral to the viral life cycle. Upon initial entry to the host, viruses encounter mucosal barriers that prevent the viral HA from attaching to sialic acid receptors found on the host cell surface (10–12). Mucosal fluids naturally express decoy receptors, such as heavily glycosylated mucins, that can neutralize virus by binding the HA before the virus has had the chance to reach the host cell (10, 13). When this occurs, the viral NA can release virus particles that have become bound to these mucosal barriers by cleaving terminal sialic acid from decoy receptor glycans, allowing the HA to reach and bind the sialic acid receptors required for attachment to host cells (10, 11). Following a successful replication cycle, virus particles begin to bud from the host cell membrane. During this process, the HA of newly formed virus particles can become reattached to sialic acids still expressed on the surface of the host cell, preventing the release of newly formed viral particles (14). However, the viral NA cleaves terminal sialic acids from the host cell, allowing the release of new viral particles (15). Finally, once viruses are released from infected cells, viral particles can form aggregates through interactions between sialylated HAs or other glycoproteins present in the mucus fluid. Individual viral particles can be released from these aggregates by the enzymatic activity of the NA (14, 16, 17).

Evidently, the NA has integral roles in the viral life cycle, from the point of first attachment to the final dispersal of nascent viral particles. Antibodies that target the NA may inhibit viral egress from infected cells and subsequent virus transmission. Indeed, vaccination regimes that induce anti-NA antibodies have been shown to induce broad protection in mice (18–23), and the presence of anti-NA immunity has been shown to significantly reduce virus shedding and morbidity in humans (24–28). Although preliminary studies successfully explored the use of NAs as vaccine candidates (21–23, 25, 29–31), the role of anti-NA antibodies in preventing influenza virus transmission is relatively undefined. Given that influenza B viruses predominantly circulate in human hosts, there is a potential to eradicate this pathogen by using vaccination regimes that severely impact transmission. Hence, in this study, we assessed anti-NA immunity, induced by a recombinant NA protein vaccine candidate, for its ability to broadly inhibit influenza B virus transmission in the guinea pig model. Therefore, our work addresses all three research areas of the National Institute of Allergy and Infectious Disease (NIAID)’s strategic plan toward a universal influenza virus vaccine (32).

## RESULTS

### Intranasal vaccination with B/Malaysia/2506/2004 NA prevents homologous transmission from vaccinated donor guinea pigs to naive recipients.

**FIG 1:**
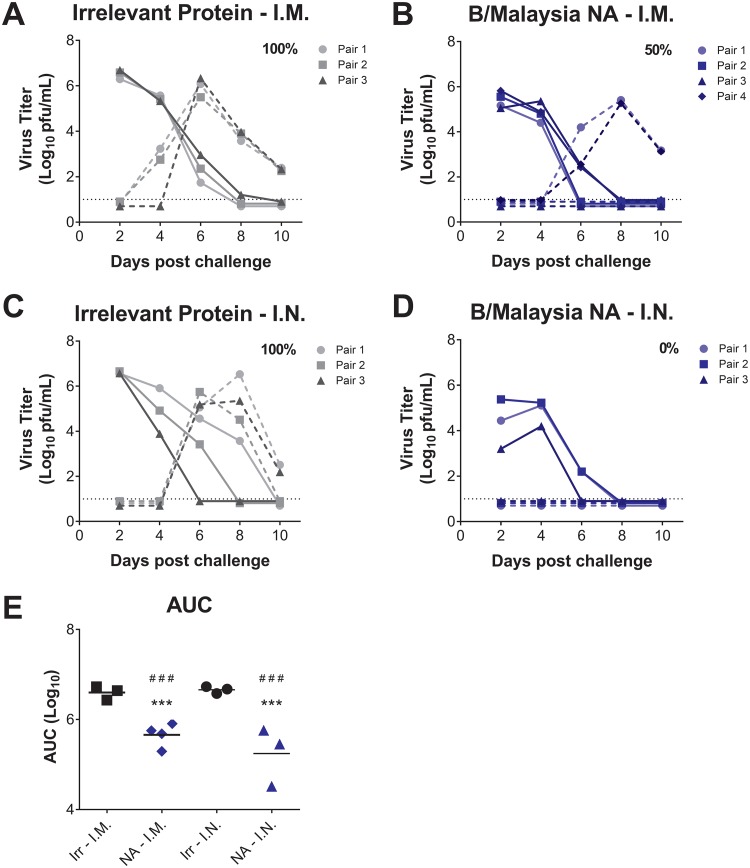
Transmission of B/Malaysia/2506/2004 influenza virus from vaccinated guinea pigs to naive guinea pigs in the airborne transmission model. Guinea pigs were vaccinated i.m. or i.n. with H7 HA (irrelevant protein) or B/Malaysia/2506/2004 NA (B/Malaysia NA). Following a prime-boost vaccination regime, vaccinated guinea pigs were i.n. challenged with B/Malaysia/2506/2004 influenza virus, and virus titers in nasal wash samples from vaccinated donors and naive recipients were assessed at days 2, 4, 6, 8, and 10 postinfection. Virus titers in irrelevant protein (A) and B/Malaysia/2506/2004 NA (B) i.m.-vaccinated donor (full line) and naive recipient (dashed line) guinea pigs were determined. Virus titers in irrelevant protein (C) and B/Malaysia/2506/2004 NA (D) i.n.-vaccinated donor (full line) and naive recipient (dashed line) guinea pigs. The percent transmission from vaccinated to naive guinea pigs is displayed in each figure panel. The dotted black line represents the limit of detection. (E) Differences in virus titers in vaccinated donors from panels A to D represented as the area under the curve (AUC). ***, *P* < 0.001 compared to i.m. irrelevant protein-vaccinated guinea pigs; ###, *P < *0.001 compared to i.n. irrelevant protein-vaccinated guinea pigs.

Preliminary studies have indicated that NA recombinant protein vaccination via the intranasal (i.n.) route leads to reduced weight loss in influenza virus-infected mice compared to mice vaccinated via the intramuscular (i.m.) route (20). In this study, we assessed the roles of systemic and mucosal immunity in preventing influenza B virus transmission in the guinea pig model of influenza virus transmission. Although guinea pigs do not display any signs of disease, they are a good model for assessing influenza virus transmission, including in the context of vaccination (33, 34). As such, we vaccinated guinea pigs here via the i.m. (systemic immunity) or i.n. (mucosal immunity) routes with either an irrelevant recombinant protein or the B/Malaysia/2506/2004 NA recombinant protein in a prime-boost vaccination regime. We then infected i.m.-vaccinated guinea pigs (vaccinated donors) with 10^4^ PFU of B/Malaysia/2506/2004 virus and compared viral titers in the nasal washes at 2, 4, 6, 8, and 10 days postchallenge. Our results showed that vaccination with B/Malaysia/2506/2004 NA resulted in a clear reduction in viral titers compared to guinea pigs vaccinated with an irrelevant control protein (Fig. 1A, B, and E). In the airborne transmission model, the transmission of B/Malaysia/2506/2004 virus was observed in all guinea pig pairs in which the donor had been vaccinated i.m. with an irrelevant protein (Fig. 1A). However, only 50% of donors vaccinated i.m. with the B/Malaysia/2506/2004 NA were able to transmit the homologous virus to their naive recipient partner (Fig. 1B).

We next assessed how vaccinating guinea pigs via the i.n. route affected influenza B virus transmission. Our results indicated that i.n. vaccination with B/Malaysia/2506/2004 NA resulted in a significant reduction in viral titers compared to guinea pigs vaccinated with an irrelevant control protein (Fig. 1C, D, and E). When we measured virus transmission from i.n.-vaccinated donors to naive recipients, transmission events were detected in all pairs in which donor animals had been vaccinated i.n. with an irrelevant protein (Fig. 1C). Remarkably, no transmission events were observed between donors vaccinated i.n. with B/Malaysia/2506/2004 NA and naive recipient partners (Fig. 1D). These results suggest that vaccination via the i.n. route can fully prevent influenza B virus transmission.

### Intranasal vaccination leads to increased anti-NA immunity at the mucosal surface.

**FIG 2:**
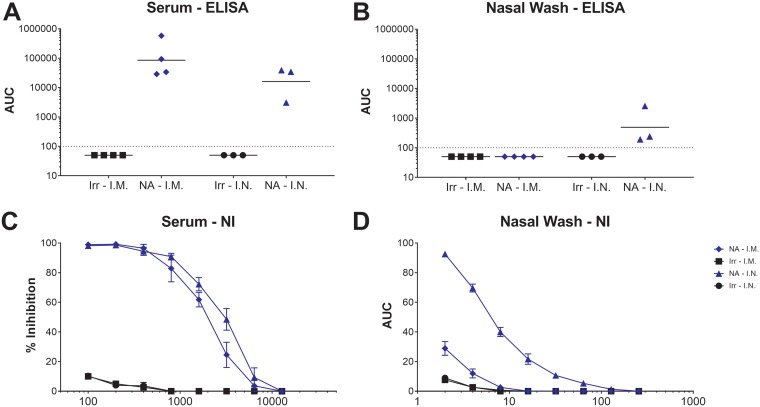
Anti-NA serological responses are increased at the mucosal site following i.n. vaccination. Guinea pigs were vaccinated i.m. or i.n. with H7 HA (irrelevant protein; labeled “Irr”) or B/Malaysia/2506/2004 NA (labeled “NA”). After a prime-boost vaccination regime, IgG antibody responses (A and B) and NA inhibition (NI) activity (C and D) were assessed in the prechallenge serum and nasal washes. The limit of detection of the enzyme-linked immunosorbent assay (ELISA) was an AUC value of 1:100. Samples that did not reach this value were assigned a value of 1:50 for graphing purposes. Error bars represent the standard error of the mean (SEM).

To dissect the reasons for the differences in transmission between i.m.- and i.n.-vaccinated donor guinea pigs, we assessed systemic and mucosal serological responses. We found no statistically significant differences in the IgG antibody responses against B/Malaysia/2506/2004 purified virus in the prechallenge sera of i.m.- and i.n.-vaccinated guinea pigs (Fig. 2A), although the serum IgG responses were slightly higher in i.m.-vaccinated animals. This result was mirrored when we assessed NA inhibition (NI) toward B/Malaysia/2506/2004 in the prechallenge serum (Fig. 2C). However, in prechallenge nasal washes, only guinea pigs vaccinated via the i.n. route had clearly detectable IgG antibody responses (Fig. 2B) and increased NA inhibition (NI) activity (Fig. 2D) compared to animals vaccinated via the i.m. route. These data suggest that vaccinating via the i.n. route leads to important mucosal immune responses that are integral for preventing influenza B virus transmission.

### Vaccination with B/Malaysia/2506/2004 NA partially prevents heterologous influenza B virus transmission from vaccinated donor guinea pigs to naive recipients.

**FIG 3:**
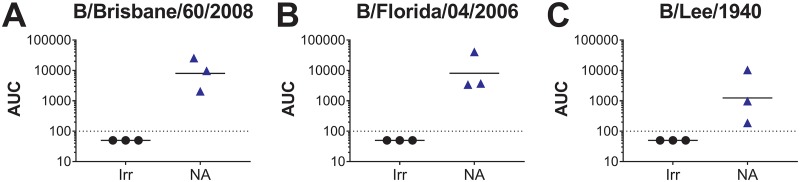
Vaccination with B/Malaysia/2506/2004 NA induces broad, anti-NA antibody responses in guinea pigs. Using a prime-boost vaccination regime, guinea pigs were immunized i.n. with H7 HA (irrelevant protein; labeled “Irr”) or B/Malaysia/2506/2004 NA (labeled “NA”). At 4 weeks after the boost, prechallenge serum was collected, and antibody responses against B/Brisbane/60/2008 (A), B/Florida/04/2006 (B), and B/Lee/1940 (C) purified viruses were assessed. The limit of detection of the assay was an AUC value of 1:100. Samples that did not reach this value were assigned a value of 1:50 for graphing purposes.

After observing that i.n.-induced immunity can fully prevent homologous influenza B virus transmission among guinea pigs, we wanted to explore the breadth of this immunity against a heterologous influenza B virus strain. It has been previously shown that vaccination with recombinant influenza B NA results in broadly protective heterologous antibody responses against both B/Yamagata/16/1988-like and B/Victoria/2/1987-like influenza B viruses in the mouse model (20). This finding was further corroborated by the existence of highly cross-reactive murine and human monoclonal antibodies that bind to conserved epitopes (4, 9). Given these data, we also wanted to assess whether i.n. vaccination with B/Malaysia/2506/2004 NA could induce broadly inhibitory anti-NA antibody responses capable of preventing heterologous influenza B virus transmission in the guinea pig model. To assess this, we collected prechallenge serum from NA-vaccinated guinea pigs and measured their antibody responses toward a panel of purified influenza B viruses. We found that guinea pigs vaccinated with B/Malaysia/2506/2004 NA had robust antibody responses toward the B/Brisbane/60/2008 virus (B/Victoria/2/1987-like lineage) (Fig. 3A) and B/Florida/04/2006 (B/Yamagata/16/1988-like lineage) (Fig. 3B) in prechallenge sera. Measurable antibody responses toward the ancestral B/Lee/1940 lineage virus were also found in prechallenge sera (Fig. 3C). These data demonstrate that vaccination with B/Malaysia/2506/2004 NA can induce broad antibody responses against heterologous influenza B virus NAs.

**FIG 4:**
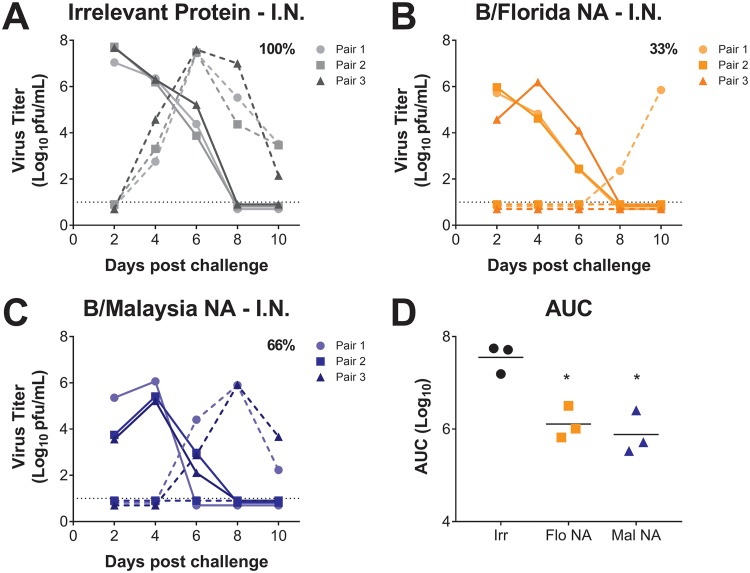
Airborne transmission of B/Florida/04/2006 influenza virus from B/Malaysia/2506/2004 NA-vaccinated guinea pigs to naive guinea pigs. Guinea pigs were vaccinated i.n. with H7 HA (irrelevant protein; “Irr” in panel D), B/Florida/04/2006 NA (homologous vaccination; “Flo NA” in panel D), or B/Malaysia/2506/2004 NA (heterologous vaccination; “Mal NA” in panel D). After a prime-boost vaccination regime, vaccinated guinea pigs were i.n. challenged with B/Florida/04/2006 influenza virus, and virus titers in the nasal wash in vaccinated donors and naive recipients were assessed at days 2, 4, 6, 8, and 10 postinfection. Virus titers in irrelevant protein (A), B/Florida/04/2006 NA (B), and B/Malaysia/2506/2004 NA (C) i.n.-vaccinated donor (full line) and naive recipient (dashed line) guinea pigs were determined. The percent transmission from vaccinated to naive guinea pigs is displayed in each figure panel. The dotted black line represents the limit of detection. (D) Differences in virus titers in the vaccinated donors from panels A to C are represented as the AUC. *, *P* < 0.05 (compared to i.n. irrelevant protein-vaccinated guinea pigs).

Next, we i.n. vaccinated guinea pigs with an irrelevant protein control, B/Florida/04/2006 NA (homologous vaccination), or B/Malaysia/2506/2004 NA (heterologous vaccination) in a prime-boost vaccination regime. At 4 weeks after the boost, we infected vaccinated guinea pigs (donors) with 10^4^ PFU of B/Florida/04/2006 and compared viral titers in the nasal washes at 2, 4, 6, 8, and 10 days postchallenge. Our results show that guinea pigs vaccinated with irrelevant protein had high levels of virus replication (Fig. 4A). However, vaccination with homologous B/Florida/04/2006 virus NA (Fig. 4B) and heterologous B/Malaysia/2506/2004 virus NA (Fig. 4C) prior to infection with B/Florida/04/2006 resulted in lower viral titers compared to guinea pigs vaccinated with an irrelevant control protein, as measured by the area under the curve (AUC) of donor nasal wash virus titers (Fig. 4D). Despite these differences in viral titers, homologous and heterologous NA vaccination did not completely abrogate airborne transmission of B/Florida/04/2006 from i.n.-vaccinated donor guinea pigs to naive recipients. Transmission of B/Florida/04/2006 from vaccinated donors to naive recipients occurred in 33% of guinea pig pairs in which the donor had been vaccinated with the homologous (B/Florida/04/2006) NA and in 67% of pairs in which the donor had been vaccinated with the heterologous (B/Malaysia/2506/2004) NA compared to 100% transmission from donors vaccinated with an irrelevant protein (Fig. 4A to C).

### Transmission from naive donors to vaccinated guinea pigs is significantly reduced and results in reduced virus replication in vaccinated recipients.

**FIG 5:**
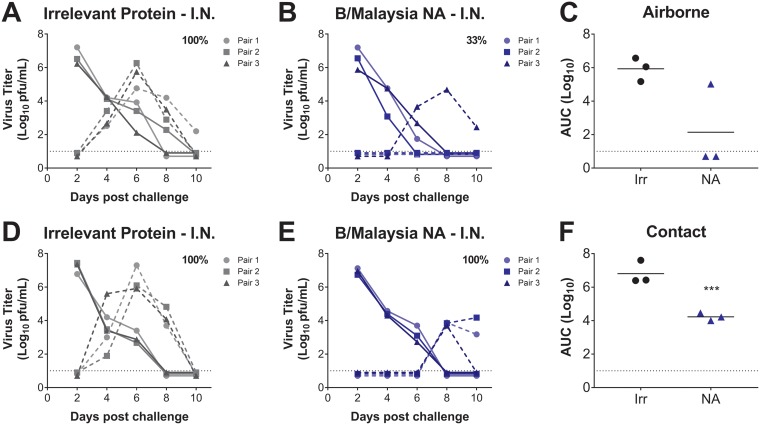
Transmission of B/Malaysia/2506/2004 influenza virus from naive to vaccinated guinea pigs in airborne and contact transmission models. (A and B) Naive guinea pigs were i.n. challenged with B/Malaysia/2506/2004 influenza virus. The following day, transmission from infected guinea pigs to guinea pigs vaccinated i.n. with H7 HA (irrelevant protein; labeled “Irr” in panels C and F) or B/Malaysia/2506/2004 NA (labeled “NA” in panels C and F) was assessed in an airborne transmission model. Virus titers in irrelevant control (A) and B/Malaysia/2506/2004 NA (B) naive donor (full line) and vaccinated recipient (dashed line) transmission pairs were determined. (D and E) Naive guinea pigs were i.n. challenged with B/Malaysia/2506/2004 influenza virus. The following day, transmission from infected guinea pigs to guinea pigs vaccinated i.n. with H7 HA (irrelevant protein) or B/Malaysia/2506/2004 NA was assessed in a contact transmission model. Virus titers in irrelevant protein (D) and B/Malaysia/2506/2004 NA (E) naive donor (full line) and vaccinated recipient (dashed line) transmission pairs were determined. The percent transmission from vaccinated to naive guinea pigs is displayed in each figure panel. The dotted black line represents the limit of detection. (C and F) Differences in virus titers in vaccinated recipients from panels A, B, D, and E are represented as the AUC. ***, *P < *0.001 (compared to i.m. irrelevant protein-vaccinated guinea pigs).

Given our observation that mucosal vaccination of donor guinea pigs with the B/Malaysia/2506/2004 NA completely prevented transmission of the homologous influenza B virus to unvaccinated recipients, we were interested in determining whether recipient guinea pigs previously i.n. vaccinated with B/Malaysia/2506/2004 NA would be susceptible to infection from naive donors via the airborne route. To this end, we inoculated naive donor guinea pigs with 10^4^ PFU of B/Malaysia/2506/2004 and assessed transmission to recipient guinea pigs that had been previously i.n. vaccinated with an irrelevant protein or B/Malaysia/2506/2004 NA. On days 2, 4, 6, 8, and 10 postchallenge, we assessed virus titers in nasal washes from both naive donors and vaccinated recipients. Airborne virus transmission was highly efficient from naive virus donors to recipients vaccinated with an irrelevant protein, occurring in all transmission pairs (Fig. 5A). However, naive donors transmitted B/Malaysia/2506/2004 virus to only one of three recipients vaccinated i.n. with the B/Malaysia/2506/2004 NA (Fig. 5B). In addition, there was a reduction in nasal wash virus titers in the one vaccinated recipient infected with B/Malaysia/2506/2004 via airborne transmission compared to those guinea pigs vaccinated with an irrelevant protein (Fig. 5C).

These data suggest that immunity induced via i.n. vaccination is partially permissive of infection via airborne transmission routes. Thus, we next determined the susceptibility of vaccinated guinea pigs to infection by naive donors in the contact model. Our results indicate that i.n. vaccination was permissive of infection in recipient guinea pigs vaccinated both with irrelevant protein and with the B/Malaysia/2506/2004 NA (Fig. 5D and E). However, there was a significant reduction in nasal wash viral titers in recipient guinea pigs vaccinated i.n. with the B/Malaysia/2506/2004 NA compared to those vaccinated with an irrelevant protein. Overall, although our data suggest that i.n. B/Malaysia/2506/2004 NA vaccination is infection permissive, it does hamper subsequent influenza B virus replication, as demonstrated by lower viral titers and a shorter duration of infection.

## DISCUSSION

In this study, we show that a vaccination regime, designed to induce mucosal immunity to the influenza virus NA, adversely affects the efficiency of subsequent influenza B virus infection and transmission in the guinea pig model. We demonstrate that prior NA vaccination of a virus-infected donor guinea pig via the i.n. route can seemingly prevent onward transmission of a homologous influenza B virus (0/3 transmission events with B/Malaysia/2506/2004 and 1/3 with B/Florida/04/2006). Interestingly, vaccination via the i.m. route reduced subsequent viral titers in infected donor animals but only partially prevented onward virus transmission to a naive recipient. Furthermore, we identified that i.n. vaccination with the NA protein induces mucosal antibody responses with increased magnitude and NA inhibitory activity compared to those induced by i.m. vaccination. Not surprisingly, serum IgG responses were slightly higher (although not significantly) in i.m.-vaccinated guinea pigs compared to those vaccinated via the i.n. route, lending support to the hypothesis that mucosal rather than systemic immunity is beneficial in preventing influenza virus transmission. Our data also demonstrate that antibodies toward the viral NA are responsible, at least in part, for the integral role that mucosal immunity plays in virus transmission from a vaccinated, infected donor onward to a naive, susceptible recipient.

Although we found that a homologous B/Malaysia/2506/2004 NA vaccination, followed by a B/Malaysia/2506/2004 challenge, inhibited transmission to naive animals, we were still able to detect heterologous virus transmission of B/Florida/04/2006 influenza virus from B/Malaysia/2506/2004 NA-vaccinated guinea pigs (Fig. 4). Despite these results, the transmission events from B/Malaysia/04/2006 NA-vaccinated donors to naive recipients were significantly reduced, as were viral titers, compared to irrelevant protein-vaccinated donors challenged with B/Florida/04/2006. Interestingly, in all of the transmission events from NA-vaccinated guinea pigs to naive recipients, we found that viral titers were reduced in the naive recipients (Fig. 1B and Fig. 4C). This may be due to the lower viral input transmitted from the vaccinated donors resulting in less viral replication and earlier clearance of infection or—more likely—a shifted peak in virus shedding that was missed with the chosen sampling time points.

Our finding that NA-vaccinated donor guinea pigs are unable to transmit influenza B virus despite relatively robust replication in the nasal cavity raises the question about the mechanism underlying transmission inhibition. One possibility is that even a slight reduction in nasal titers can adversely impact transmissibility of influenza viruses, which is a plausible though unexciting explanation. However, virus titers in animals receiving i.m. or i.n. vaccination were similar, and only i.n. vaccination blocked transmission effectively (Fig. 1). A second explanation is that antibodies inhibiting the enzymatic activity of the viral NA alters virus transmissibility. It has been reported that in the absence of NA activity, influenza viruses tend to aggregate (14), which may negatively impact transmissibility. In addition, virus might be trapped by decoy receptors on natural defense proteins like mucin, also leading to aggregation and reduced ability to transmit. Finally, it is possible that virus is efficiently released from an infected host and transmitted to a susceptible one, but that the virus is coated by anti-NA antibody that impairs its ability to initiate a new infection, becoming trapped by mucins in the respiratory tract of the recipient. Regardless of the mechanism of action, however, mucosal immunity against the viral NA seems to be key in preventing efficient interhost transmission; our study and others (34, 35) suggest that serum antibody levels are less important in this specific regard.

In our studies, we also performed transmission experiments in which naive, influenza B virus-infected donor guinea pigs were paired with recipients previously vaccinated i.n. with the homologous NA. Interestingly, we observed inefficient transmission to one of three vaccinated recipients in the airborne model and efficient transmission to three of three vaccinated recipients in the contact model. However, in both models, the vaccinated guinea pigs that were infected by transmission demonstrated very low nasal wash virus titers and a short duration of shedding.

In humans, several observations have been made regarding the impact of anti-NA antibody titers on virus infection and disease. In influenza A virus (H3N2) challenge studies, individuals with high anti-NA antibody titers were permissive to infection with the homologous influenza virus, but they had reduced nasal virus shedding and illness after infection (25, 27). Similar observations were made in a more recent human challenge study with pandemic H1N1 virus (28). In addition, NI titers have been shown to correlate with protection from natural infection with H1N1 and H3N2 infection, respectively, in two independent studies (24, 26). Anti-NA antibodies might also have played a role in protecting H2N2 experienced individuals during the H3N2 pandemic in 1968 (27). Thus, in humans, anti-NA antibodies might confer a degree of protection from natural infection with influenza A viruses, while in challenge studies they do not confer immunity from infection but do reduce viral shedding and disease. While no comparable studies exist for influenza B, the influenza A data in humans echo our findings in the guinea pig model using influenza B virus. When NA-vaccinated guinea pigs were directly inoculated with influenza B virus, they showed reduced nasal wash virus titers, similar to that which has been shown in human challenge studies. When NA-vaccinated guinea pigs were the recipients, paired with naive, infected donors (analogous to natural infection in humans), we observed only partial inhibition of transmission. However, a reduction in the magnitude and duration of virus replication in NA vaccinated recipients that became infected by transmission was observed, a finding reflected in human studies. What remains unknown in humans is the extent to which anti-NA antibodies affect the contagiousness of influenza virus-infected individuals, i.e., are humans with robust anti-NA antibody titers less likely to transmit influenza viruses on to other people? This question, while difficult to answer with human studies, is very important from a public health perspective. If anti-NA immunity impairs the ability of infected individuals to infect others, then vaccines inducing high titers of anti-NA antibodies would be valuable for control of influenza B on a population scale. Our data from the guinea pig model suggest that vaccinated animals (those with mucosal antibodies inhibiting the enzymatic function of the viral NA) are poor onward transmitters to susceptible partner animals, thus breaking the transmission chain.

Potentially, there are many ways to translate our findings into vaccines and vaccination regimens that induce robust anti-NA immunity. The easiest solution might be NA-only vaccines that are given in addition to the current vaccines or admixture of recombinant NA to inactivated vaccines (to avoid an additional shot) (3, 20). The use of mucosally administered inactivated vaccines is currently being explored, and recombinant NA could be added to those vaccines as well (36). In addition, next-generation influenza virus vaccines, e.g., based on RNA or DNA vaccines or viral vectors, could also include NA as an antigen (37, 38).

In conclusion, our data suggest that anti-NA immunity can significantly contribute to reduction of influenza B virus shedding. Importantly, mucosal anti-NA immunity blocks efficient transmission of influenza B viruses in the guinea pig model. In theory, vaccines that impair the transmission efficiency of influenza B viruses among humans could, with sufficient herd immunity, drastically limit their spread. This is an especially attractive proposition for influenza B viruses, which lack an animal reservoir, because it opens up the possibility of their eradication from the human population. These data argue for further research into and development of influenza virus vaccines inducing robust mucosal anti-NA antibody responses.

## MATERIALS AND METHODS

### Ethics statement.

All animal procedures in this study were performed in accordance with the Institutional Animal Care and Use Committee (IACUC) guidelines and have been approved by the Icahn School of Medicine at Mount Sinai IACUC.

### Cells and viruses.

Sf9 cells (CRL-1711, ATCC) for baculovirus rescue were grown in Trichoplusia Ni medium-formulation Hink insect cell medium (Gemini Bioproducts) supplemented with 10% fetal bovine serum (FBS; Sigma) and penicillin (100 U/ml)-streptomycin (100 μg/ml) solution (Gibco). BTI-TN-5B1-4 (High Five) cells for protein expression were grown in serum-free SFX medium (HyClone) supplemented with penicillin (100 U/ml)-streptomycin (100 μg/ml) solution. Madin-Darby canine kidney (MDCK) cells were grown in Dulbecco modified Eagle medium supplemented with 5% FBS and penicillin (100 U/ml)-streptomycin (100 μg/ml) solution. The influenza B virus strains B/Malaysia/2506/2004, B/Florida/04/2006, B/Brisbane/60/2008, and B/Lee/1940 were grown in 10-day-old embryonated chicken eggs (Charles River) for 72 h at 33°C. The eggs were then cooled overnight at 4°C before harvesting the allantoic fluid. Harvested allantoic fluid was centrifuged at 4,000 × *g* for 10 min at 4°C to remove debris. Viruses were then aliquoted and stored at –80°C prior to determining stock titers via plaque assay. To purify viruses for enzyme linked immunosorbent assays (ELISAs), virus stocks were grown in eggs (as above) and harvested 72 h later. Virus was then purified over a 30% sucrose cushion in 1× NTE buffer (0.5 mM NaCl, 10 mM Tris-HCl [pH 7.5], 5 mM EDTA), and the purified virus concentration was determined using Bradford’s reagent.

### Recombinant proteins.

Soluble HA and NA proteins containing a T4 foldon trimerization domain or a vasodilator stimulated phosphoprotein tetramerization domain, respectively, were generated using the baculovirus expression system as previously described (9, 20, 39). The HA and NA recombinant proteins expressed C-terminal and N-terminal hexahistidine tags, respectively, for purification purposes.

### Guinea pig vaccination.

Five- to six-week-old female guinea pigs were purchased from Charles River Laboratory and randomly assigned to different vaccination groups. Guinea pigs were either primed i.m. or i.n. with 10 μ of A/Shanghai/1/2013 H7 HA (as an irrelevant protein control), B/Malaysia/2506/2004 NA, or B/Florida/04/2006 NA recombinant protein adjuvanted with 10 μ of poly(I⋅C) (Invivogen). A boost via the i.n or i.m. routes with 10 μ of poly(I⋅C)-adjuvanted recombinant protein was given 28 days later. At 4 weeks after the boost, the vaccinated guinea pigs were used as vaccinated donors or recipients in transmission experiments.

### Transmission experiments.

Guinea pig transmission experiments were performed in an environmentally controlled chamber (model 6030; Caron Products & Services, Inc.), as previously described (40). Donor guinea pigs were anaesthetized with ketamine (30 mg/kg) and xylazine (5 mg/kg) before being challenged i.n. with 10^4^ PFU of B/Malaysia/2506/2004 or B/Florida/04/2006 in 300 μ of phosphate-buffered saline (PBS). The following day, donor and recipient transmission pairs were set up in cages that precluded physical contact but allowed lateral airflow (airborne transmission) or were cocaged (contact transmission). On days 2, 4, 6, 8, and 10 from the initial donor challenge, nasal washes were collected from anaesthetized donor and recipient guinea pigs. Blood was collected via venipuncture of the lateral saphenous vein at the prime, challenge, and euthanasia time points for the determination of serum antibody responses and NA inhibition (NI). Nasal washes were also collected at prime and challenge time points for the determination of antibody responses and NI. On days 18 to 21 postchallenge, guinea pigs were anaesthetized with ketamine (44 mg/kg) and xylazine (5 mg/kg) and terminally bled.

For B/Malaysia/2506/2004 transmission experiments, guinea pigs were housed at 20°C and 20% relative humidity (RH). The B/Florida/04/2006 transmission experiments occurred at 5°C and 20% RH. These environmental settings have been previously shown to be optimal for these influenza B virus strains to transmit efficiently among guinea pigs (41).

### Virus titers.

Virus titers were determined by plaque assay on MDCK cell monolayers. Virus stocks and nasal washes were diluted 10-fold in PBS and incubated on MDCK cells for 1 h before the addition of an agarose overlay containing a final concentration of 0.64% agarose (Oxoid), 1× minimum essential medium (MEM) (10% 10× minimal essential medium [Gibco], 2 mM l-glutamine, 0.1% of sodium bicarbonate [wt/vol; Gibco], 10 mM 4-HEPES, 100 U/ml penicillin–100 μ/ml streptomycin, and 0.2% bovine serum albumin (BSA), 1 μ/ml TPCK (tolylsulfonyl phenylalanyl chloromethyl ketone)-treated trypsin, and 0.1% (wt/vol) DEAE (diethylaminoethyl)-dextran was added to the cells. The cells were then incubated for 72 h at 33°C, and visible plaques were counted after fixing them with 3.7% formaldehyde and visualization with a crystal violet counterstain (Sigma-Aldrich). All virus titers are presented as the log_10_ PFU/ml. The limit of detection for these assays was 10 PFU/ml. The area under the curve (AUC) values for virus titers over the time course of infection were calculated using the log PFU/ml, and the AUC value was calculated using GraphPad Prism 7 software.

### Enzyme linked immunosorbent assay.

Immulon plates (Immulon 4HBX; Thermo Scientific) were coated with 2 μg/ml of purified virus (50 μl/well) in coating buffer (KPL coating solution; Sera Care) at 4°C overnight. The following day, the plates were washed three times with PBS containing 0.1% Tween 20 (PBS-T) and blocked in blocking solution (3% goat serum and 0.5% milk in PBS-T) for 1 h at room temperature. After blocking, prediluted serum was added to the first well to a final concentration of 1:100 in blocking solution. For assessing antibody responses in the nasal washes, a neat nasal wash was added to the first well. Serum and nasal washes were then serially diluted and incubated at room temperature for 2 h. Plates were then washed three times with PBS-T before the addition of donkey anti-guinea pig IgG-horseradish peroxidase (IgG-HRP; EMD Millipore) in blocking solution for 1 h at room temperature. Plates were then washed four times with PBS-T with shaking and then the *O*-phenylenediamine dihydrochloride (OPD) substrate (SigmaFast OPD; Sigma-Aldrich) was added. After 10 min of incubation at room temperature, the reaction was stopped by adding 50 μl of 3 M HCl to the mixture. The optical density (OD) was measured at 490 nm on a Synergy 4 plate reader (BioTek). A cutoff value of the average of the OD values of blank wells plus three standard deviations was established for each plate and used for calculating the AUC values of the nasal washes and the sera, which were the readout for this assay.

### Enzyme-linked lectin assay to determine NI.

Immulon plates (Immulon 4HBX; Thermo Scientific) were coated with 50 μ/μ of fetuin (150 μl/well) in coating buffer (KPL coating solution; Sera Care) at 4°C overnight. The following day, the plates were washed six times with PBS-T and blocked in blocking solution (5% BSA in PBS) for 2 h at room temperature. While the plates were being blocked, virus was serially diluted 1:2 on a separate 96-well plate in PBS containing 1% BSA. After this blocking step, 100 μ of serially diluted virus was transferred to the fetuin-coated plates, and the plates were incubated at 33°C for 2 h. The plates were then washed, and a secondary solution of peanut agglutinin (PNA) conjugated to HRP (5 μ/ml) was added to the plates. After a 2-h incubation in the dark, the plates were washed six times and developed with 100 μ of SigmaFast OPD. After developing for 7 min, 50 μ of 3 M HCl was added, and the OD was measured at 490 nm on a Synergy 4 plate reader (BioTek).

To perform NI assays, Immulon plates were coated and blocked as described above. While the plates were being blocked, guinea pig serum and nasal washes were serially diluted 1:2 in a separate 96-well plate in PBS. A starting dilution of 1:50 was used for serum, while we started with a neat nasal wash. A 75-μ portion of virus diluted to a 2× 50% effective concentration was added to each well of the serially diluted serum plate, and the plates were shaken at room temperature for 2 h. Before the shaking incubation time expired, the fetuin-coated plates were washed six times with PBS-T, and 100 μ of the virus/serum mixture was transferred to the fetuin-coated plates, followed by incubation for 2 h at 33°C. The remainder of the NI assay was performed as described above. The values obtained from the plate reader were divided by the average of the virus-only well subtracted from the no-virus control well and multiplied by 100 to obtain the NA activity. The percent inhibition was calculated by subtracting the NA activity from 100.

### Statistics.

Grouped data (except in Fig. 2C and D) are expressed as individual dot plots, and means are represented as geometric mean (GEM). Data in Fig. 2C and D are presented as grouped data, and means are expressed as standard errors of the mean (SEM). Statistical differences between two groups were determined by Student paired *t* test. Statistical differences between three or more groups were determined by two-way analysis of variance, followed by a Bonferroni multiple-comparison test. All statistical analyses were performed using GraphPad Prism 7 for Windows. In all cases, probability levels of <0.05 were indicative of statistical significance (*, *P* < 0.05; ***, *P* < 0.001).
